# Evidence supporting vertical transmission of *Salmonella* in dairy cattle

**DOI:** 10.1017/S0950268815002241

**Published:** 2015-09-30

**Authors:** D. L. HANSON, G. H. LONERAGAN, T. R. BROWN, D. J. NISBET, M. E. HUME, T. S. EDRINGTON

**Affiliations:** 1 International Center for Food Industry Excellence, Texas Tech University, Lubbock, Texas, USA; 2 United States Department of Agriculture, Agricultural Research Service, Southern Plains Agricultural Research Center, Food and Feed Safety Research Unit, College Station, Texas, USA

**Keywords:** Epidemiology, *Salmonella*, zoonoses

## Abstract

We set out to investigate whether *Salmonella enterica* could be recovered from various tissues of viable neonatal calves immediately following parturition. Eleven samples were aseptically collected from each of 20 calves and consisted of both left and right subiliac and prescapular lymph nodes (LN), mesenteric LN, spleen and liver, as well as intestinal tissue (including luminal contents) from the small intestine, caecum, spiral colon and rectum. In addition, a faecal sample was collected from 19 of the dams. *Salmonella* was recovered from at least one sample from 10 of the 20 neonates. Across all calves, *Salmonella* was recovered from 12·7% of all samples and from LN in particular, *Salmonella* was recovered from 10·0%, 5·0%, and 5·0% of subiliac, prescapular, and mesenteric LN, respectively. Within calves, *Salmonella* was recovered from 0% to 73% of samples and across tissues, estimates of *Salmonella* prevalence were greatest in the caecum (30%) but was never recovered from the right pre-scapular LN. These data provide evidence of vertical transmission from a dam to her fetus such that viable calves are born already infected and thereby not requiring faecal–oral exposure for transmission. This new knowledge ought to challenge – or at least add to – existing paradigms of *Salmonella* transmission dynamics within cattle herds.

Dairy food products and the cattle from which they are derived are frequently identified as vehicles and reservoirs for human exposure to *Salmonella enterica*, respectively [1–4]. The burden of *Salmonella* in cattle populations appears to be meaningfully greater, or even ubiquitous, in cattle housed in the southern climes of the United States [3]. Because of its shared importance to public and animal health, considerable effort has been invested to understand and, if possible, control both the transmission of *Salmonella* and the pathogenesis of salmonellosis in dairy cattle [5–7]. At the centre of current understanding and, therefore, models of transmission dynamics of *Salmonella* within herds is the assumption of fecal–oral transmission.

It has been reported that primary exposure occurs early in life in that shedding has been observed in calves as young as 1 day old [7]. More recently, we examined calves within a few hours of birth and routinely recovered *Salmonella* from rectal swabs (T. S. Edrington, unpublished observations). Our findings, in addition to the frequent recovery of *Salmonella* from neonates [7], led us to speculate that *Salmonella* might in fact transmit vertically from the dam to her fetus *in utero*. If so, calves might already be infected with *Salmonella* at birth rather than post natum. Such vertical transmission would saliently change – or add to – our understanding of transmission dynamics, and would provide challenges for the design of effective control strategies given that attempts to prevent exposure after birth might be too late to prevent initial infection. Therefore, the objective of this study was to investigate whether *Salmonella enterica* could be recovered from various tissues of viable – apparently healthy – neonatal calves immediately post natum.

Sample collection occurred during a 1-day period in August and then again in September (2014) on a commercial, dry-lot dairy located in the Southern High Plains of the United States. During these sampling periods, a convenience sample of 20 Holstein cows were enrolled at the time of parturition. Enrolment was based on a cow beginning parturition while the samplers were present on the dairy, parturition progressed normally, and the cows appeared healthy. Twenty animals were selected to provide sufficient power to detect at least one positive sample given a design prevalence of 2·5% with 95% confidence. Because no reports have been published of *Salmonella* prevalence in newly born, healthy calves, we arbitrarily selected a design prevalence of 2·5% in this pilot study. Within 2 min post-parturition, calves were humanely euthanized following standard operating procedures using a captive bolt. Following euthanasia, calves were placed on a disinfected surface for sample collection. Eleven samples were aseptically collected from each of 20 calves and consisted of both left and right subiliac and prescapular lymph node (LN), mesenteric LN, spleen and liver, as well as tissue and luminal contents from the small intestine, caecum, spiral colon and rectum. In addition to the samples collected from the calves, a faecal-grab sample was collected from 19 of the 20 dams prior to parturition. Between each set of sample collections (and between the samples collected from the dam and its calf), new disposable gloves were used and equipment was disinfected. Samples were aseptically collected and placed into individual plastic bags. Sample-containing bags were sealed and samples were transported on wet ice to the USDA-ARS laboratory in College Station, Texas, for bacterial culture and characterization.

All samples were cultured quantitatively and qualitatively for *Salmonella* within 48 h of collection. LN were cultured as previously described [8]. Briefly, tetrathionate broth (80 ml) was added to each sample bag containing the trimmed and surface-sterilized LN and mixed for 60 s. For quantitative estimation, 1 ml of pulverized LN/tetrathionate broth mixture was removed and applied to selective agar (Petrifilm^™^ EB, 3M Health Care, USA) in duplicate and incubated overnight (37 °C). Films with bacterial growth were transferred to xylose lysine deoxycholate (XLD) plates containing 10 *µ*g/ml cefsoludin and 15 *µ*g/ml novobiocin and incubated (37 °C, 24 h). Black colonies were counted and converted to log_10_ c.f.u./g LN tissue. Following initial plating for quantification, the LN/tetrathionate mixture was incubated overnight (37 °C). One hundred microlitres were then transferred to 3 ml Rappaport–Vassiliadis (RV) broth and incubated at 42 °C for 24 h and then plated on Brilliant Green agar supplemented with 80 *µ*g/ml sulphadiazanine (BGA_s_). Plates were incubated (37 °C overnight). Liver, spleen, and gastrointestinal samples were cultured as above with the exceptions that the sample was neither trimmed nor surface-sterilized prior to processing, 5 ml RV broth was used, and post-enrichment samples were plated on BGA plates supplemented with 25 *µ*g/ml novobiocin (BGA_nov_). Fecal samples from the dam were processed by inoculating 10 g faeces into 90 ml tetrathionate and 50 *µ*l of the mixture was plated onto XLD agar using a commercially available spiral plater (Spiral Biotech Autoplate 4000; Advanced Instruments, USA) and incubated (37 °C, 24 h). Up to three morphologically typical colonies from each positive sample (XLD, BGA_nov_, BGA_s_) were serogrouped using slide agglutination with *Salmonella* antiserum (Difco Laboratories, USA). Antimicrobial susceptibility was determined using broth microdilution methods according to the manufacturer's directions (Trek Diagnostic Systems, USA). Minimum inhibitory concentrations were calculated and breakpoints were set using those established by the Clinical and Laboratory Standards Institute (CLSI) [9]. Where CSLI breakpoints were not available, those used by the National Antimicrobial Resistance Monitoring System were used [10]. *E. coli* ATCC 25 922, *E. coli* ATCC 35 218, and *E. faecalis* ATCC 29 212 were used as quality control organisms. *Salmonella* isolates (or a portion thereof) were further characterized by serogrouping, denaturing gradient gel electrophoresis (DGGE) to achieve a predicted serotype [11, 12], and through a classical serotyping scheme by the National Veterinary Services Laboratory (NSVL, Ames, USA). At least one isolate from each positive sample was subjected to DGGE. Where more than one serogroup was identified in a sample, an isolate representing each serogroup was subjected to serotype prediction for a total of 140 isolates characterized by DGGE. A portion of those isolates (*n* = 30) were sent to NVSL in order to evaluate the concordance the DGGE and classical serotyping methods. Of those samples not serotyped by either method (*n* = 35, i.e. they were of the same serogroup within a sample), serotype was assumed from information derived from DGGE and/or classical serotyping performed on other isolates within the sample. Proportions (and confidence intervals for proportions) were calculated both manually and with the aid of a commercially available spreadsheet (Microsoft Excel for Mac 2011, version 14.5.4; Microsoft Corp., USA).

Two hundred and twenty samples were collected from 20 neonates over two sample collections in August (*n* = 88) and September (*n* = 132). *Salmonella* was recovered from at least one sample in 50% of neonates (Fig. 1). Of all neonatal tissue and dam faecal samples, *Salmonella* was recovered from 12·7% [*n* = 28/220; 95% confidence interval (CI) 8·6–17·9] and 94·7% (*n* = 18/19, 95% CI 74·0–99·9), respectively. *Salmonella* was recovered from 10·0% (*n* = 4/40, 95% CI 2·8–23·7), 5·0% (*n* = 2/40, 95% CI 0·6–16·9), and 5·0% (*n* = 1/20, 95% CI 0·1–24·9) of subiliac, prescapular, and mesenteric LN (Fig. 2), respectively. Further, *Salmonella* was recovered from 15·0% (*n* = 3/20, 95% CI 3·2–37·9) of spleen and liver samples. From rostral to caudal, the prevalence estimates of *Salmonella* recovery from gastrointestinal samples were 5·0% (*n* = 1/20, 95 CI 0·1–24·9), 30·0% (*n* = 6/20, 95% CI 11·9–54·3), 20·0% (*n* = 4/20, 95% CI 5·7–43·7) and 20·0% (*n* = 4/20, 95 CI 5·7–43·7) for the small intestine, caecum, spiral colon and rectum, respectively. Of the 220 samples 1·8% (*n* = 4, 95% CI 0·5–4·6) contained *Salmonella* above the limit of quantification. These samples included one caecum sample (1·8 log_10_ c.f.u./g sample), two rectum samples (1·2 and 2·6 log_10_ c.f.u./g sample) and one prescapular LN (1·8 log_10_ c.f.u./g LN).

**Fig. 1.**
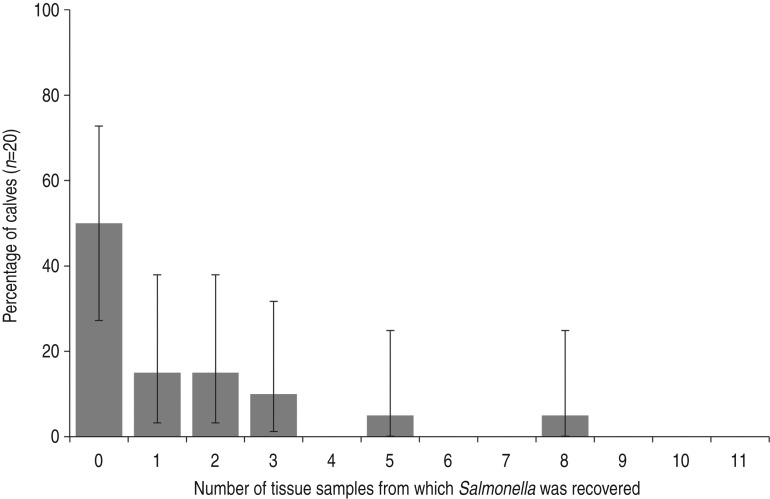
Percentage of calves by the number of tissue samples from which *Salmonella* was recovered; error bars represent 95% confidence limits.

**Fig. 2.**
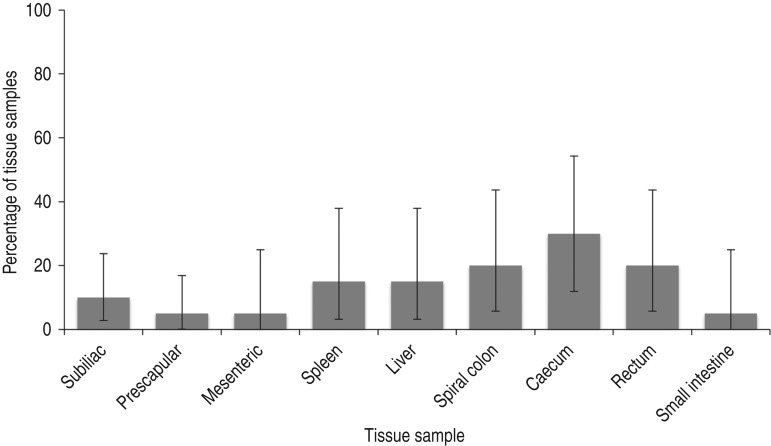
Observed prevalence of *Salmonella* by calf tissue type; error bars represent 95% confidence limits.

Serogrouping was conducted on 138 isolates derived from 46 positive samples (three isolates per sample). Of the 46 positive samples, 21·7% (*n* = 10, 95% CI 10·9–36·4) contained multiple serogroups within the same sample. Of these multi-serogroup samples, 90% (*n* = 9) and 10% (*n* = 1) had two and three serogroups, respectively. Within the remaining samples from which *Salmonella* was recovered, *n* = 36 indistinguishable serogroups were observed. Of all isolates, serogroups consisted of C_1_ (*n* = 58, 42·0%), C_2_ (*n* = 41, 29·7%), E_1_ (*n* = 21, 15·2%) and other (*n* = 18, 13·0%; Supplementary Table S1).

Multiple serogroups were observed in nine faecal samples collected from the dams and one spleen sample collected from a calf. Seven different combinations of serogroups were identified when multiple serogroups were present in a sample. The same serogroup was identified in 40% (*n* = 4) of the 10 animals from which *Salmonella* was recovered from both the dam and the calf. Distinct serogroups were identified in the remaining six (60%) instances where *Salmonella* was recovered from both the dam and calf.

Ten different serotypes were identified across the 138 isolates (Supplementary Table S2). One isolate could only be distinguished as either *S.* Typhimurium or *S.* Anatum var. 15+. *S.* Montevideo was the most frequently isolated serotype from both dam and calf. *S.* Muenchen was the second and third most prevalent serotype identified in calf tissues and dam faeces, respectively. *Salmonella* serotypes Newport, Mbandaka, Senftenberg and Typhimurium were identified in cow faeces but never in any of the calf tissues. Conversely, *S.* Cerro and *S.* Kentucky were recovered only from calf tissues (Supplementary Table S2). In only one dam-calf pair (calf 1; Supplementary Table S2), all of the serotypes observed in the calf were indistinguishable from those observed in the dam. In five instances where a single serotype was identified across the calf samples, it was distinct from that recovered from the dam. Multiple serotypes were observed in four of the calves and six of the dams (where both were positive; Supplementary Table S3) and in another four dams when only the dam was *Salmonella* positive (data not shown). *Salmonella* serotype did not appear to correspond with the calf tissue from which it was cultured. All isolates derived from neonatal samples were pansusceptible to the panel of drugs against which they were tested. Where resistance was detected, it was of the MDR-AmpC phenotype and observed exclusively in *S.* Newport (serogroup C2), and was only observed in 11 isolates all of which were recovered from dams.

Herein we provide compelling evidence for vertical transmission of *Salmonella* from the dam to fetus. Such a finding might not be unexpected in cases of clinical disease and septicaemia as has been observed in events of human listeriosis. Further, cases of vertical transmission of *Salmonella* in dams have been reported but saliently, have resulted in fetal death and typically, clinical disease in the dam [13]. However, the dams included in our study were apparently asymptomatic carriers, and most interestingly, the calves were visually healthy and had they not been euthanized for the purposes of this research, they would have been expected to grow and thrive similarly to other calves on the dairy. While the enrolled cows appeared healthy and experienced a routine parturition, no clinical or bacteriological follow-up was performed other than routine herd-health observation. It is possible that these dams may have been early in the pathogenesis of salmonellosis; if so, however, this was not reported to the authors during frequent follow-up farm visits.

In those calves from which *Salmonella* was recovered, the typical number of tissues per calf that yielded at least one isolate was 2·8. *Salmonella*, therefore, was commonly recovered from calves and when it was recovered, was frequently recovered from various tissues within apparently healthy calves. This finding of asymptomatic infection within the gastrointestinal tract of dams and within various tissues of their fetuses raises salient questions of the interaction of *Salmonella* with the bovine host. By this we mean that some *Salmonella* infections – including extraintestinal infections – appear to occur in the absence of gross pathological changes or clinical disease unlike many infections with *S*. Dublin for example. Moreover, in our other work, we have observed frequent carriage of *Salmonella* in peripheral LN collected at slaughter from animals that have passed all ante-mortem and post-mortem inspections [14]. Knowledge of how this intracellular pathogen interacts and potentially evades the host immune system is needed. This becomes particularly important in the context of efforts to reduce or eliminate *Salmonella* from cattle herds. If *Salmonella* does evade effective immunological recognition, novel approaches to interrupt within- and between-animal dissemination are needed. Vertical transmission further adds challenges to control efforts. That is, approaches designed to prevent newborn calves from exposure to *Salmonella* through such practices as hospital pen management, separating dams from calves, or colostrum management will have only limited success in preventing infection if a substantial proportion are born already infected.

We have not determined when during gestation *Salmonella* infect fetuses. Development of fetal immunity is thought to occur during the last trimester of gestation [15]. An interesting possibility is that if *Salmonella* infect fetal tissues during this development, the fetus might fail to recognize *Salmonella* as non-self in a similar manner by which animals become persistently infected with bovine viral diarrhoea virus [16]. If so, the factors *Salmonella* use to evade the immune system might help explain the ubiquitous and generally asymptomatic burden of *Salmonella* cattle herds of the Southern High Plains. It might be that *in utero* infection during immunological development results in the loss of – or an impaired – ability to immunologically recognize *Salmonella* as a foreign organism.

In the present study we did not explore whether or not the *Salmonella* recovered from the calves originated from systemic dissemination within the dam. It is possible that prior intrauterine infection – such as prior metritis or iatrogenically introduced during artificial insemination – was the source of the *Salmonella* infection for the fetus. If so, then presumably the conceptus and then the fetus may have been exposed to *Salmonella*. This potential localized source, however, requires that conception, embryonic development and implantation occur in a *Salmonella*-infected uterus, and that the *Salmonella* infection within the fetus persists to parturition.

While we believe the most likely phenomenon that explains our observations is vertical transmission (whether from either a systemic or localized infection), alternative explanations are also possible. It is possible that calves were infected during parturition and *Salmonella* subsequently and rapidly disseminated to various tissues. This does not seem plausible – at least it is likely that this possibility does not fully account for our observations – in that *Salmonella* was recovered from diverse tissues such as peripheral LN, liver and spleen. Moreover, we euthanized the calves within 2 min – mostly much sooner – following birth. A further alternative explanation is that cross-contamination during tissue collection contributed to our observations. Specifically to avoid this possibility to the practical extent possible, calves were placed onto a disinfected surface for necropsy, and samples were collected using aseptic techniques and then placed into sterile containers. Moreover, cross-contamination, if it did occur, would fail to explain recovery of *Salmonella* from LN in that once they were received in the USDA-ARS laboratory, they were trimmed and surface-sterilized using methods developed and validated to remove surface contamination [17]. Furthermore, previously we have recovered *Salmonella* from rectal swabs of calves within hours (sometimes within an hour) after birth. In addition, differences in serotypes from the dams and their fetuses were routinely observed in that serotypes were only occasionally shared; as such, cross-contamination from the faeces of dams to their fetuses seems unlikely. We believe, therefore, that despite the manifest challenges of conducting necropsies on a commercial dairy where sterility was not practically possible, cross-contamination – if it occurred – was certainly not the primary factor that contributed to our observations. An approach to further explore and describe the extent of vertical transmission of *Salmonella* could include movement of periparturient cows from commercial dairies to a suitably clean location and deliver the calf by caesarean section. While this approach seems plausible, the simple movement (and, therefore, stress of movement), could influence the likelihood of *Salmonella* dissemination through the tissues of the dam and presumably, its fetus.

Herein we provide what we believe is compelling evidence for vertical transmission from a dam to her fetus *in utero*. If so, *Salmonella* transmission from one animal to another might occur without the need for fecal–oral transmission. This new knowledge ought to fundamentally challenge our preconceptions of *Salmonella* transmission dynamics, its interaction with the host immune system, and approaches to control. New ecological models that capture this route of transmission are clearly warranted.

The authors assert that all procedures contributing to this work comply with the ethical standards of the relevant national guides on the care and use of animals used in research. Procedures involving animals were reviewed and approved by the Texas Tech University Institutional Animal Care and Use Committee (protocol 14033-04).

## SUPPLEMENTARY MATERIAL

For supplementary material accompanying this paper visit http://dx.doi.org/10.1017/S0950268815002241.
